# Seroprevalence of Pertussis in the Netherlands: Evidence for Increased Circulation of *Bordetella pertussis*

**DOI:** 10.1371/journal.pone.0014183

**Published:** 2010-12-01

**Authors:** Sabine C. de Greeff, Hester E. de Melker, Pieter G. M. van Gageldonk, Joop F. P. Schellekens, Fiona R. M. van der Klis, Liesbeth Mollema, Frits R. Mooi, Guy A. M. Berbers

**Affiliations:** 1 Epidemiology and Surveillance, Centre for Infectious Disease Control, National Institute for Public Health and the Environment, Bilthoven, The Netherlands; 2 Laboratory for Infectious Diseases and Screening, National Institute for Public Health and the Environment, Bilthoven, The Netherlands; 3 Laboratory for Infectious Diseases, Groningen, The Netherlands; Columbia University, United States of America

## Abstract

**Background:**

In many countries, the reported pertussis has increased despite high vaccination coverage. However, accurate determination of the burden of disease is hampered by reporting artifacts. The infection frequency is more reliably estimated on the basis of the prevalence of high IgG concentrations against pertussis toxin (IgG-Ptx). We determined whether the increase in reported pertussis in the last decade is associated with an increase in the number of infections.

**Methodology/Principal Findings:**

In a cross-sectional population-based serosurveillance study conducted in 2006-07, from a randomly selected age-stratified sample of 7,903 persons, serum IgG-Ptx concentrations were analyzed using a fluorescent bead-based multiplex immuno assay. In 2006-07, 9.3% (95%CI 8.5-10.1) of the population above 9 years of age had an IgG-Ptx concentration above 62.5 EU/ml (suggestive for pertussis infection in the past year), which was more than double compared to 1995-96 (4.0%; 95%CI 3.3-4.7). The reported incidence showed a similar increase as the seroprevalence between both periods.

**Conclusions:**

Although changes in the vaccination program have reduced pertussis morbidity in childhood, they have not affected the increased infection rate in adolescent and adult pertussis. Indeed, the high circulation of *B. pertussis* in the latter age-categories may limit the effectiveness of pediatric vaccination.

## Introduction

**Figure 1:**
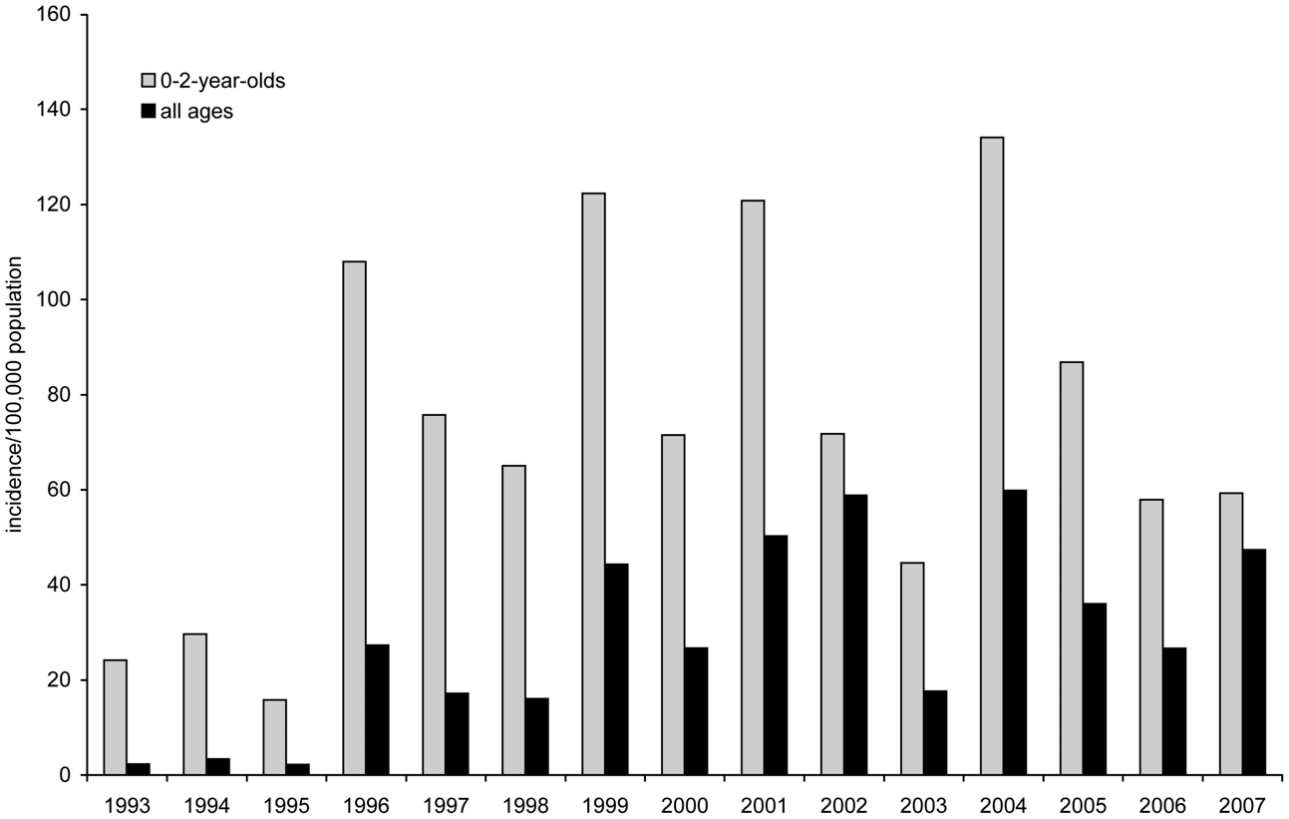
Incidence per 100,000 population of reported pertussis cases in the Netherlands in 1993–2007, for 0-2-year-olds and for all ages.

In the last decades, an increase of the reported incidence of clinical pertussis cases has been observed in many countries despite a high vaccination coverage [1], [2], [3], [4], [5]. Various explanations have been given for the pertussis re-emergence, including increased awareness, improved diagnostics, waning of vaccine-induced immunity and adaptation of the causative pathogen *Bordetella pertussis* [6], [7], [8]. In the Netherlands, despite a consistently high vaccination coverage for decades [9], increased numbers of pertussis notifications have been observed since 1996 with epidemic peaks every 2–3 years (Figure 1) [1], [2]. The majority (95%) of these reported pertussis cases are serologically confirmed. A whole-cell pertussis vaccine has been used in the Netherlands since 1953 and this is administered in the National Immunization Programme (NIP) to infants at 2, 3, 4 and 11 months. The coverage of the NIP is circa 96% [9]. In 2001 a booster vaccination with acellular vaccine for 4-year olds was introduced and in 2005, the whole cell vaccine was replaced by an acellular vaccine [1]. Previously, we reported that the sudden increase in pertussis notifications in 1996 was largely due to a genuine increase in clinical pertussis cases and could only partly be explained by changes in diagnostic practice [2]. The increase in reported pertussis cases may be due to a higher circulation of the causative organism *B. pertussis* and/or to an increase in the fraction of infections which lead to clinical symptoms.

At present, estimations of the true circulation are most reliably made based on prospective studies in defined populations [10] and on the basis of the prevalence of high IgG concentrations against pertussis toxin (IgG-Ptx) in the population. In response to an infection with *B. pertussis* almost all patients show an increase in IgG against pertussis toxin which reaches a maximum within a few weeks. This increase is followed by a steady decline during 6–12 months after infection [11], [12]. Ptx is expressed only by *B. pertussis* and cross-reacting antigens have not been described. Previously, we showed that an IgG-Ptx level of at least 100 local U/ml (which is equal to 125 EU/ml [13]) is a highly specific criterion for recent pertussis infection [14]. Interpretation of IgG-Ptx concentrations is complicated by the fact that all pertussis vaccines contain Ptx. The Dutch whole-cell vaccine hardly induced IgG-Ptx antibodies. On the contrary, vaccination with acellular pertussis vaccines induces high concentrations of IgG-Ptx, but these rapidly wane within the first 6 months [15], [16], [17]. This is true for both primary vaccination in infancy and the booster vaccination at four years of age.

In the current study we aimed to estimate the age-specific seroprevalence of pertussis infections in a cross-sectional sero-survey of the Dutch population in February 2006- July 2007. Based on serodiagnostic cut-off levels of IgG-Ptx we estimated what percentage of the population experienced a recent pertussis infection and what factors are associated with a high IgG-Ptx concentration. To further improve our understanding of the changes in the epidemiology of pertussis over the last decade we compared the age-specific seroprevalence with results obtained from a similar national survey conducted in 1995–1996 [14], [18] and with incidence rates calculated from mandatory notifications in both periods. Our results show that, although the changes in the vaccination program have reduced pertussis morbidity in childhood, they have not affected the increased infection rate in adolescent and adult pertussis. Indeed, the high circulation of *B. pertussis* in the latter age categories may limit the effectiveness of pediatric vaccination.

## Methods

The study proposal was approved by the Medical Ethics Testing Committee of the foundation of therapeutic evaluation of medicines (METC-STEG) in Almere, the Netherlands (clinical trial number: ISRCTN 20164309) and all participants provided signed informed consent for blood sampling and/or a questionnaire. If participants were minors consent was obtained from two parents or guardians.

### Study population and design

Between February 2006 and July 2007 a cross-sectional population-based serosurveillance study was conducted to estimate for the national population and for different subgroups the age-specific seroprevalence of antibodies against diseases targeted by the national immunization program (NIP) in the Netherlands. Details on study design and data collection were described previously [19]. Briefly, from 40 randomly (with probability to their size) selected municipalities in the Netherlands an age-stratified selection of individuals (classes 0, 1–4, 5–9,…75–79 years) was invited to give a blood sample, to complete a questionnaire and to bring their certificates from the national immunisation program. To assess the seroprevalence in migrants separately, oversampling was performed by inviting a random sample of non-Western migrants aged 0–79 years from 12 municipalities included in the nationwide sample. Similarly, oversampling was performed in eight municipalities with low immunisation coverage to assess the seroprevalence in low vaccination communities (LVCs) and in individuals who refuse vaccination based on religious grounds [19]. In total, for 6,385 participants from the national sample (including 645 individuals in the oversampled migrant group) and 1,518 individuals from areas with low vaccination coverage a serum sample was obtained and a questionnaire was completed.

Blood samples were centrifuged (10 min at 2500 rpm) and sera were stored at −80°C until analysis. With the questionnaire information was collected on demographic characteristics, socioeconomic status, educational level, household composition, and vaccination history. Furthermore, participants were asked whether and when they had had a period of coughing attacks that lasted more than two weeks in the past twelve months, and whether a clinician had diagnosed pertussis infection in the past.

### Laboratory methods

Serum antibody concentrations were analyzed as described previously [20]. In short, total IgG antibodies directed against *B. pertussis*, diphtheria and tetanus (DTaP combo vaccine) were measured simultaneously using a fluorescent bead-based multiplex immuno assay (DTaP MIA) (Luminex xMAP technology). Analysis was performed with a Bio-Plex 200 in combination with Bio-Plex manager software (Bio-Rad Laboratories, Hercules, CA). The MIA showed a high correlation with the FDA ELISA [20] in a large panel of sera with a broad range of concentrations (n = 120, Pearson's correlation coefficient  = 0.972, with log(MIA) = 0.9775*log(FDA ELISA)+0.0691). Serum values for *B. pertussis* were assigned in EU/ml [21], [22] as the in-house reference used was calibrated against the U.S. Reference Pertussis Anti-serum Human lot 3 (CBER/FDA).

### Sero-survey 1995-1996

The study design and data collection of the sero-survey conducted in 1995–1996 have been published elsewhere [14], [18], and are comparable to the survey in 2006–2007. Sera from the national sample (n = 7,735) collected from October 1995 until December 1996 were assayed in 1997 in the routine setting of the serology laboratory of the RIVM. IgG-Ptx was measured by enzyme-linked immunosorbent assay (ELISA) as previously described [14], [23]. The IgG-Ptx assay has an upper limit of detection of 500 U/ml and the lower detection limit of the assay is 5 U/ml. Results were expressed in “local” U/ml.

To enable a proper comparison between the two sero-surveys, 217 samples with a broad range of concentrations from persons of all age-groups from the 1995-96 survey were retested in the MIA. Concentrations were log-transformed and the Bland–Altman plot demonstrated good agreement between both methods. Due to the use of different references (as reflected by in-house ELISA local 100 U/ml equals 125 FDA ELISA EU/ml [13]) the MIA showed on average 1.28 times higher concentrations (paired t-test with 95%CI 1.14–1.43). There was a good correlation (with Pearson R = 0.890) between the in-house ELISA (X) and the MIA (Y) with log(Y) = 0.0911+1.0118 log(X). This equation was used to transform concentrations measured with the ELISA making them comparable to concentrations obtained with the MIA in the 2006-07 survey. All analyses were further performed with these transformed values.

### Statistical analyses

Anti-Ptx IgG levels for the national sample in both sero-surveys (excluding the oversampling of migrants in the 2006-07 survey) were divided into four categories according to the estimated average time since infection [24]: 0–20 EU/ml, 20 to <62.5 EU/ml, 62.5–125 (infection in the last 6–12 months) and ≥125 EU/ml (infection in the last 6 months). To produce seroprevalence estimates with 95% confidence intervals for the Dutch population, each person was assigned a sampling weight that incorporated the probability of selection and included adjustment for age, gender, urbanization degree and ethnicity. Since acellular vaccination may affect the IgG-Ptx immune response in recently vaccinated individuals, results are presented separately for persons targeted with the acellular vaccine (i.e. born after 1998 and below ten years of age) and those who were not.

To gain insight into the circulation of pertussis and the level of underreporting, weighted seroprevalence was compared amongst both sero-surveys and with the incidence rates calculated from mandatory notifications. Comparisons are presented as risk ratios (RR) were presented with 95% confidence intervals. Details on the mandatory notification system [1], [2] have been described previously.

For the whole 2006–2007 survey (n = 7,903), we determined by means of logistic regression analysis, whether there are risk factors for adolescents and adults that are associated with an increased chance on acquiring pertussis infection. Variables with a p-value <0.1 in the univariate model were included in the multivariate model. Backward selection was used to identify covariates that were independently associated with presumptive pertussis infection in the past year. Odds ratios (ORs) for the complete case analysis were calculated and presented with 95% confidence intervals (95% CI). Analyses were performed using Microsoft Excel and SAS version 9.1.3.

## Results

### Seroprevalence in children ≤9 years of age

**Figure 2:**
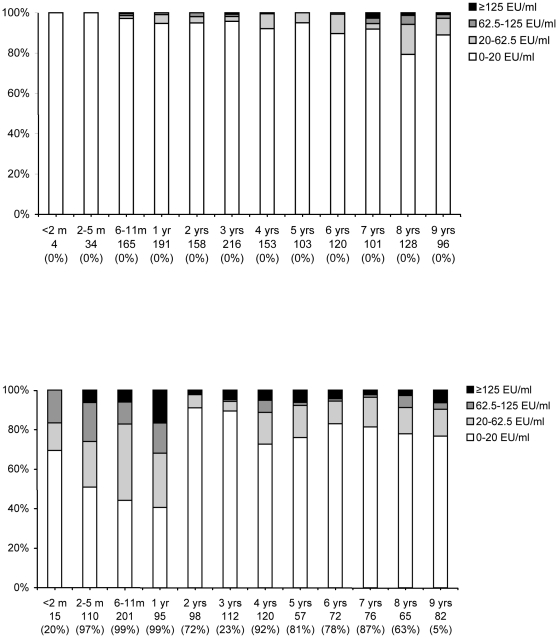
Age-specific seroprevalence of IgG-Ptx concentrations in children 0–9 years of age in 1995-96 (upper figure) and in 2006-07 (lower figure). Note: on the x-axis the age-group, number tested and in brackets the percentage targeted by the acellular vaccine are indicated. In 2006-07 children below 4 years of age could have been primed with either whole-cell or acellular vaccine in infancy (nationwide coverage circa 96%), and children 4–9 years of age could have been primed with whole-cell vaccine and may have received a preschool booster with acellular vaccine (nationwide coverage circa 90%).

Presumably due to the introduction of an acellular booster vaccination for 4-year olds in 2001 and the replacement of the whole cell vaccine by an acellular vaccine in 2005, we observed that children in 2006-07 had generally higher concentrations of IgG-Ptx than children in the 1995-96 survey (Figure 2). In the era of acellular vaccine, 32% (21–43) of the 1 year old children had an IgG-Ptx concentration ≥62.5 EU/ml, compared to 0.9% (0-2) in 1995-96. However, high levels of IgG-Ptx decreased by age within a year: only 2% (CI 0-5) of the 2-year olds had an antibody concentration ≥62.5 EU/ml in 2006-07, while 91% (86–96) had a concentration below 20 EU/ml. Due to the booster, 11% (6–16) of the 4-year olds had an IgG-Ptx concentration ≥62.5 EU/ml and this decreased with age to 4% (0–7) in 7-year olds. In 8- and 9-year olds the fraction with a concentration ≥62.5 EU/ml was increased again to 9% (3–15) and 10% (4–16), respectively.

### Seroprevalence in persons >9 years of age

**Figure 3:**
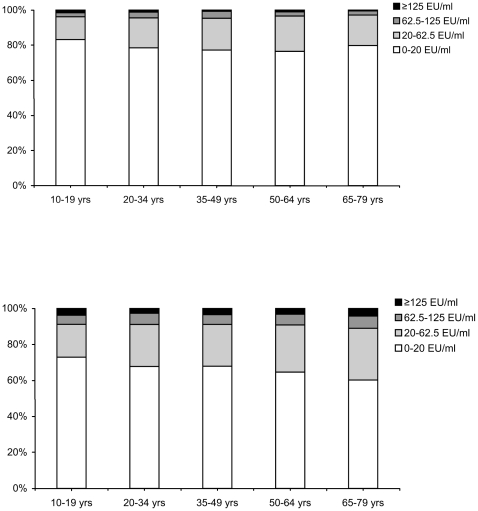
Age-specific seroprevalence of IgG-Ptx concentrations in individuals >9 years in 1995-96 (upper figure) and in 2006-07 (lower figure).

**Figure 4:**
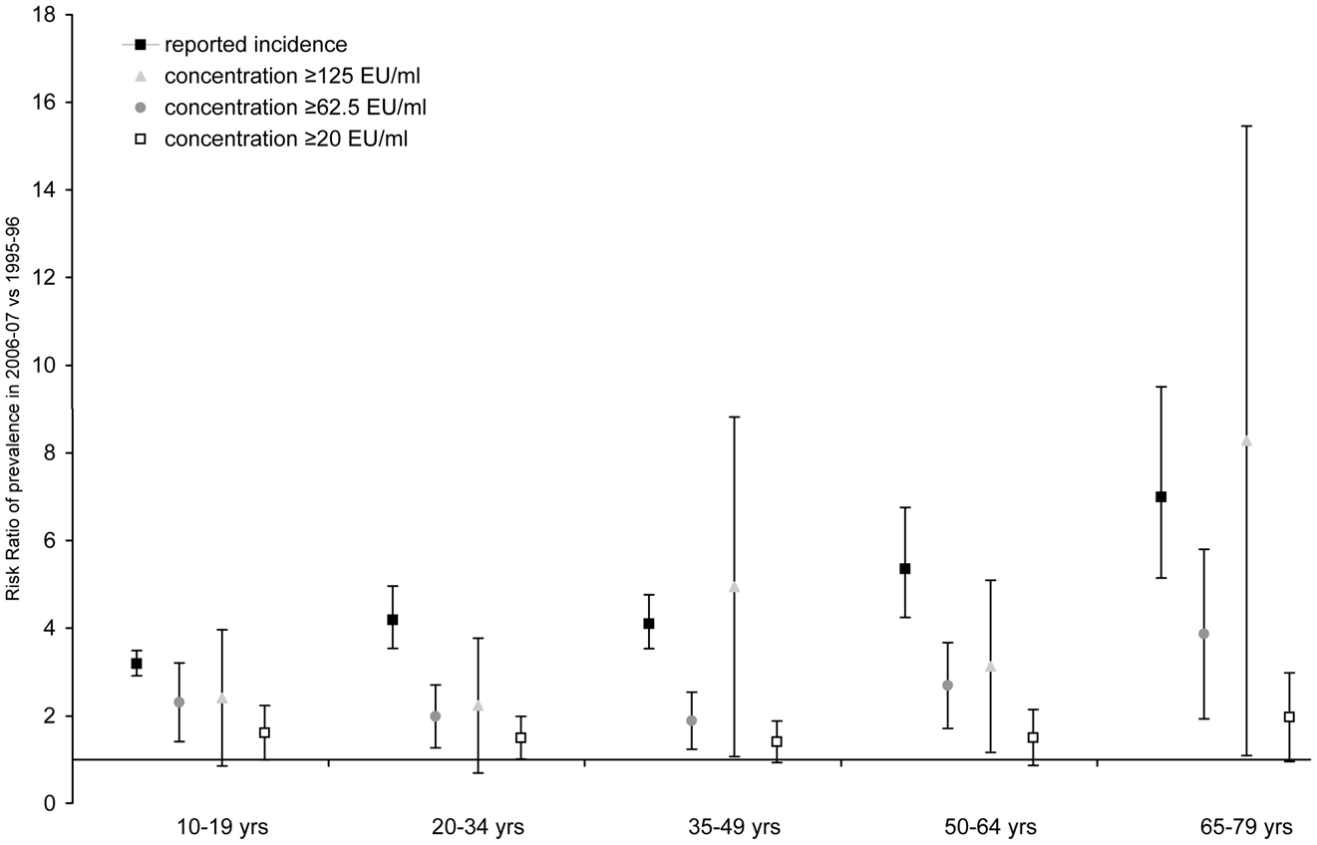
Risk ratios and 95% confidence intervals for the comparison of seroprevalence and reported incidence of pertussis in individuals >9 years in 2006-07 vs. 1995-96.

Figure 3 shows the age-specific seroprevalence based on IgG-Ptx in the national sample in 1995-96 and in 2006-07 in adolescents and adults >9 years of age. Overall, 4.0% (3.3–4.7) of the people >9 years in 1995-96 had an IgG-Ptx concentration ≥62.5 EU/ml (presumptive pertussis infection in the past twelve months) and 1.0% (0.7–1.2) ≥125 EU/ml. In 2006-07 these percentages were 9.3% (8.5–10.1) and 3.4% (2.8–3.9), respectively. Overall, the seroprevalence of concentrations ≥125 EU/ml (presumptive pertussis infection in the past six months was in 1995-96 a factor 116 and in 2006-07 a factor 100 higher than the reported incidence at the time. Figure 4 shows the age-specific risk ratios for the comparison of the seroprevalence rates between both sero-surveys and for the comparison of the reported incidence during the two sero-surveys. Per age-groups the reported incidence was 3 to 7 times higher in 2006-07 than in 1995-96. Interestingly, the prevalence of individuals with an antibody concentration ≥125 EU/ml showed similar increases, except for the 20–34 and 50–64 year olds where this increase was less pronounced.

The prevalence of reported coughing among people with an IgG-Ptx concentration ≥62.5 EU/ml increased from 17% (11–24) in 1995-96 to 25% (20–29) in 2006-07 (table 1). Especially among 35–49 year-olds and 65–79 year-olds an increase was seen. In the 2006-07 survey 19 persons (0.4%) reported to be diagnosed with pertussis in the past year, 8 of them had an IgG concentration ≥62.5 EU/ml. In 1995-96, 8 (0.1%) persons were diagnosed with pertussis in the past year; none had an IgG-Ptx concentration ≥62.5 EU/ml.

**Table 1:** Prevalence (%) of coughing symptoms in the past year according to age in individuals with IgG-Ptx concentration≥62.5 EU/ml in 1995-96 and in 2006-07.

	IgG-Ptx ≥62.5 EU/ml	
	1995-96	2006-07
**10–19 years**	26 (13–38)	24 (13–35)
**20–34 years**	20 (7–34)	28 (20–36)
**35–49 years**	5 (0–13)	21 (12–30)
**50–64 years**	27 (12–43)	17 (8–26)
**65–79 years**	16 (2–31)	36 (27–44)
**total**	17 (11–24)	25 (20–29)

With the multivariate regression model including gender, age, income and religion, it was found that male compared to female, 65–79 year olds compared to adolescents, people with low incomes compared to high income groups, and people from Reformed Congregations compared to people without religious background, were more likely to have an IgG-Ptx concentration ≥62.5 EU/ml (table 2).

**Table 2:** Risk factors for an IgG-Ptx concentration >62.5 EU/ml in individuals >9 years in 2006-07 (n = 5830).*

		No (%) with Ptx concentration ≥62.5 EU/ml	Crude OR (95% CI)	Adjusted OR (95% CI)
**Gender**	**Male**	287 (11)	Ref	
	**Female**	293 (9)	0.8 (0.7–0.9)	0.8 (0.7–0.9)
**Age**	**10–19 yrs**	81 (9)	Ref	
	**20–34 yrs**	128 (10)	1.1 (0.8–1.4)	1.1 (0.8–1.5)
	**35–49 yrs**	109 (9)	1.0 (0.7–1.3)	1.0 (0.8–1.4)
	**50–64 yrs**	118 (9)	1.0 (0.7–1.3)	1.0 (0.8–1.4)
	**65–79 yrs**	144 (12)	1.4 (1.0–1.8)	1.4 (1.0–1.9)
**Ever vaccinated**	**No**	139 (11)	Ref	
	**Yes**	190 (9)	0.8 (0.7–1.0)	
	**Unknown**	251 (11)	1.0 (0.8–1.2)	
**Living in urban area**	**No**	227 (11)	Ref	
	**Yes**	353 (9)	1.2 (1.0–1.4)	
**Ethnicity**	**Dutch or western migrant**	531 (10)	Ref	
	**Turkish + Moroccan**	16 (12)	1.3 (0.7–2.1)	
	**Other non-western migrant**	33 (9)	0.9 (0.6–1.4)	
**Persons in household**	**1–2**	286 (10)	Ref	
	**3–4**	203 (10)	1.0 (0.8–1.2)	
	**≥5**	91 (10)	1.0 (0.8–1.2)	
**Household member ≤4 years**	**No**	73 (10)	Ref	
	**Yes**	507 (10)	1.0 (0.8–1.3)	
**Number of rooms in the house**	**1–2**	32 (11)	Ref	
	**3–6**	425 (10)	0.9 (0.6–1.3)	
	**≥7**	123 (9)	0.8 (0.5–1.2)	
**Net monthly income per household**	**⩽ € 3,050**	386 (10)	1.5 (1.2–2.0)	1.5 (1.1–1.9)
	**> € 3,050**	64 (7)	Ref	
	**Unknown**	130 (10)	1.5 (1.2–2.1)	1.5 (1.1–2.1)
**Educational level** **	**Low**	81 (11)	Ref	
	**Middle**	308 (10)	0.9 (0.7–1.1)	
	**High**	191 (10)	0.8 (0.6–1.1)	
**Religion**	**No religion**	188 (10)	Ref	
	**Reformed Congregations**	75 (13)	1.4 (1.0–1.8)	1.3 (1.0–1.7)
	**Other**	317 (10)	1.0 (0.8–1.2)	0.9 (0.8–1.1)

*for 179 persons (3%) data on one or more variables was missing.

**in children below 14 years the mothers highest educational level was asked; low  =  no education or primary education, middle =  junior technical school, lower general or intermediate vocational secondary education, high =  higher vocational or higher general secondary education, pre-university or university education.

## Discussion

In this national randomized sero-survey we found that in 2006-07 approximately 9% of the population in the Netherlands above 9 years of age had a pertussis infection in the past year. This percentage has more than doubled compared to a decade ago. The increased seroprevalence is consistent with the steady increase in reported clinical cases and hospitalized cases in adolescents and adults in the past decade [1], illustrating that the observed increase in pertussis is not only due to improved reporting rate or better awareness but rather to a real increase in the circulation of pertussis. About a quarter of the adolescents and adults with presumptive pertussis infection reported to have had at least two weeks of coughing symptoms in the preceding year. These findings support the concept of endemic, though often mitigated, pertussis among adolescents and adults, in a country with a high coverage childhood vaccination program.

Increased levels of IgG-Ptx induced through vaccination with acellular pertussis vaccines cannot be distinguished from high levels due to infection and the high seroprevalence in children below 8 years of age in the 2006–2007 survey are likely to be induced by vaccination and not by natural infection [16], [25]. However, the increase with age from 7 years onwards in the proportion of children with an IgG-Ptx concentration ≥62.5 EU/ml suggests that natural infection may occur again two years after the pre-school booster. As whole cell vaccines hardly induce IgG-Ptx antibodies [17], [26], high levels measured in the group of participants older than 9 years and not eligible for the acellular vaccine are most likely due to recent infection.

The seroprevalence of presumptive infections deduced from the 1995-96 survey was similar to that reported in other countries with high vaccination coverage [10], [27]. Compared to 1995-96, the seroprevalence in 2006-07 had increased with a factor of almost 3 in adolescents and adults, and up to 8 in elderly. Similar increases were observed among 35–59 years old adults in Australia in a comparison of two serological studies performed in 1998 and 2002 [28]. In Sweden, re-introduction of pertussis vaccination in childhood decreased the circulation in children, however in Swedish adolescents aged 14–18 years and in elderly >65 years the proportion of persons with an IgG-Ptx concentration indicative for recent infection became higher suggesting an increased circulation of *B. pertussis* in older age categories [29].

We propose the increase in the circulation of *B. pertussis* can be attributed to a combination of waning immunity and the emergence of new strains [7], [8], [30]. In the Netherlands, the use of an imperfect whole cell vaccine in the beginning of the nineties may have increased the level of circulation in the population [31]. Concurrently, a change in circulating *B. pertussis* strains was observed [32]. Recently, it was shown that the strains circulating since the end of the 1990s contain a mutation which confers increased pertussis toxin which may enhance infection of primed hosts [30]. Consistent with this, we observed that the increase in reported clinical cases was more pronounced than the increase in seroprevalence, and besides, a larger part – though not statistically significant – of the presumptive infections resulted in clinical symptoms according to the questionnaire in the second survey. The upward trend by age of the proportion of presumptive pertussis in adults may indicate that the more immunity has waned the higher the chance to become infected by *B. pertussis*. In children, however, after introduction of a preschool booster and the replacement of the Dutch whole cell vaccine by an acellular vaccine, a decrease in the incidence of pertussis in children was observed, suggesting improved protection of the cohorts born after 1998 [1].

The higher seroprevalence in low income groups may be related to lower hygiene measures. Interestingly, people from Reformed Congregations - who are known to refuse vaccination for religious reasons - had higher rates of infection, possibly due to the absence of vaccine-induced immunity.

We acknowledge there are some limitations of the current study. First of all, a major concern could be the use of different laboratory techniques to measure IgG-Ptx levels. The 1995–1996 samples were tested with the RIVM ELISA, while the 2006–2007 sera were tested in a multiplex immuno-assay (MIA). However, accounting for use of different references, the results obtained with the in-house ELISA in 1995-96 turned out to be similar when tested with the MIA. Noticeably, for international comparison, the values generated with the MIA showed a high correlation with the FDA ELISA [20]. Secondly, it should be noted that this is not a longitudinal study with two time points but a comparison between two cross-sectional studies. Considering the periodicity in pertussis with peaks every 2–3 years [33], the incidence and seroprevalence in 2006-07 may be at the high end of the normal range burden in the past decade. However, based on the similarity between the increase in the prevalence of a concentration ≥125 U/ml and the reported incidence, it may be assumed that both gradually increased during the years in between the two surveys. Finally, from April 1997 a single serum sample above the diagnostic cut-off was included in the case definition for notification [2]. This means that from that time a higher proportion of patients could be notified which may partly explain the increase in reported pertussis, though not in seroprevalence.

Our results show that, although the improvements in the vaccination program have reduced pertussis morbidity in childhood, they have not affected the increase in adolescent and adult pertussis. The high circulation rates of *B. pertussis* in the latter age categories may have even limited the effectiveness of pediatric vaccination. High circulation of *B. pertussis* among adults - which is the compound effect of waning immunity and pathogen adaptation - may support the idea of introducing adult booster vaccinations [34]. However, investing in an expensive and labor-intensive recurring vaccination program for adults to boost their immunity seems paradoxical when under the current situation natural boosting takes place frequently and results in relatively mild infections in adults. When transmission remains high, mild infections among adults will be frequent, boosting clinical immunity and hence low levels of severe disease may be maintained [35]. We therefore believe it would be more efficient to invest in the development of improved vaccines which induce long lasting immunity to reduce the pertussis disease burden. For the short term however, the high circulation emphasizes the need for good protection of unvaccinated infants who are at highest risk for severe pertussis. Vaccinating people in close contact with infants, will be an (cost)effective strategy to prevent transmission of pertussis to infants [36].
